# Supplementary material for: Diversity of Antibiotic Resistance genes and Transfer Elements-Quantitative Monitoring (DARTE-QM): a method for detection of antimicrobial resistance in environmental samples
Source: Commun Biol. 2022 Mar 17;5:216. doi: 10.1038/s42003-022-03155-9 (PMC8931014; doi:10.1038/s42003-022-03155-9)
Supplement: Supplementary file 2 — Supplementary Information [file 42003_2022_3155_MOESM2_ESM.pdf]

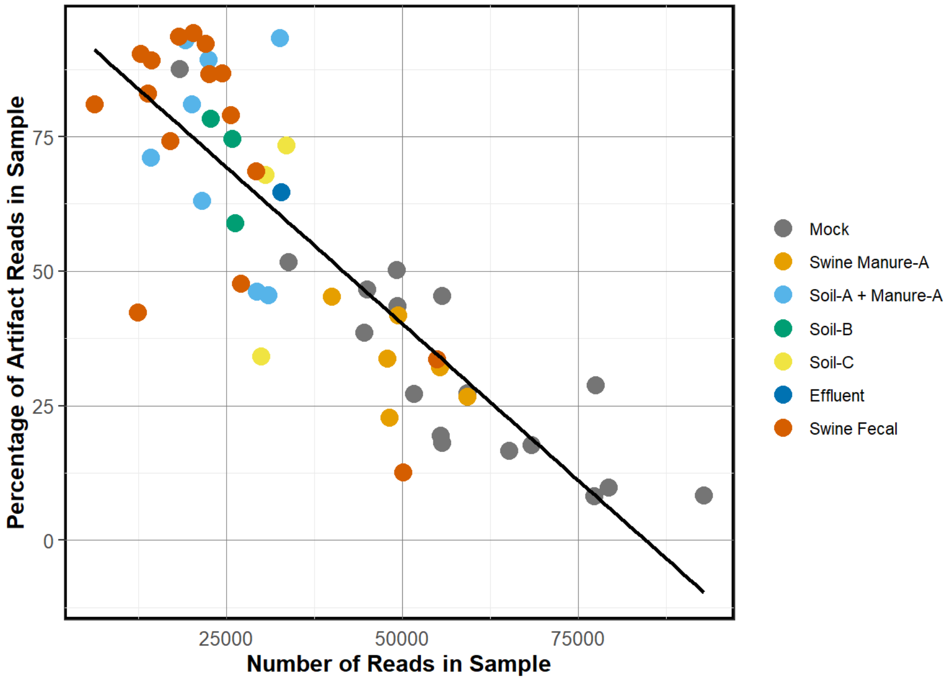

Supp Figure 1. Linear correlation of the percentage of artifacts reads present in a sample to the total number of reads in the sample. Reads were defined as sequencing artifacts if a primer was located on the 5’ end of the sequences and the read did not align to any of reference ARGs or any other location in the mock-community genomes. The percentage of sequencing artifacts observed was higher for environmental samples relative to mock community samples and was also inversely correlated (R^2 = 0.68) to the number of reads in a sample.

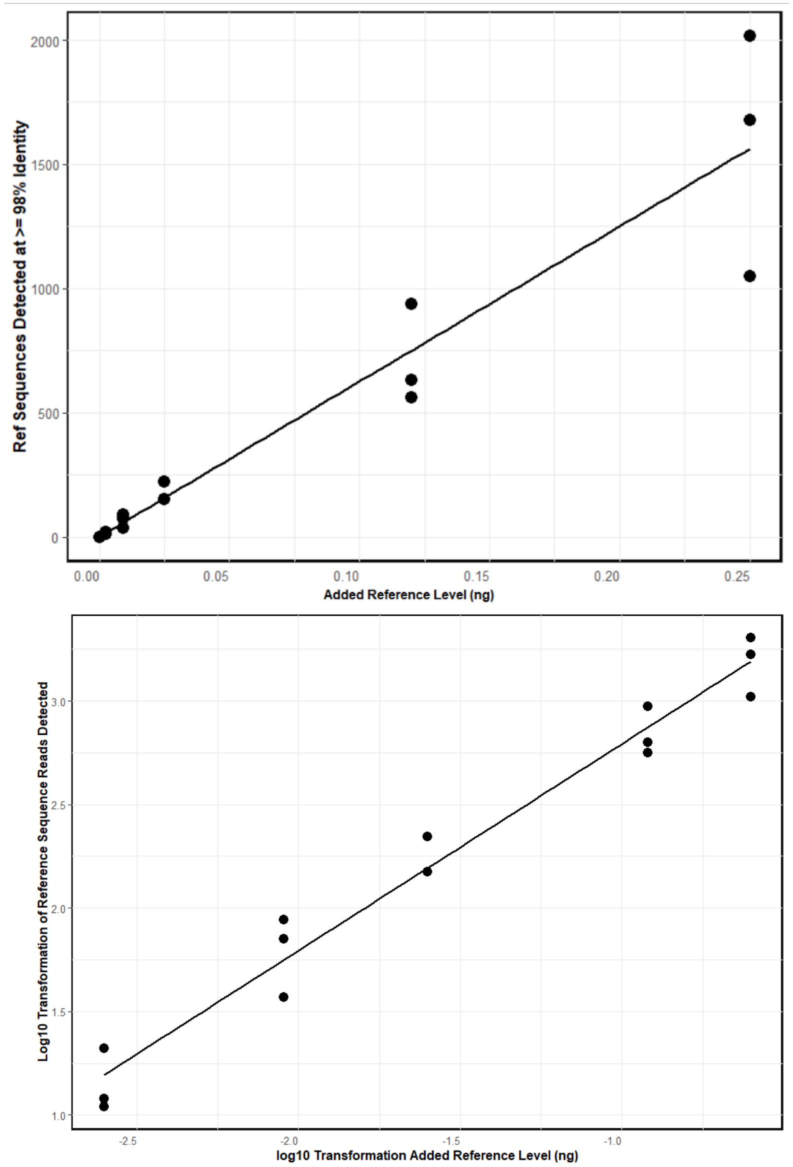

Supp Figure 2. Linear correlation between the concentration of the reference sequence added to mock community samples and the number of reads which aligned to the reference sequence. The linear model found there there to be a strong correlation (R^2 = 0.91), indicating DARTE-QM iwas sensitive to DNA quantity.

A. *erm35* alignment

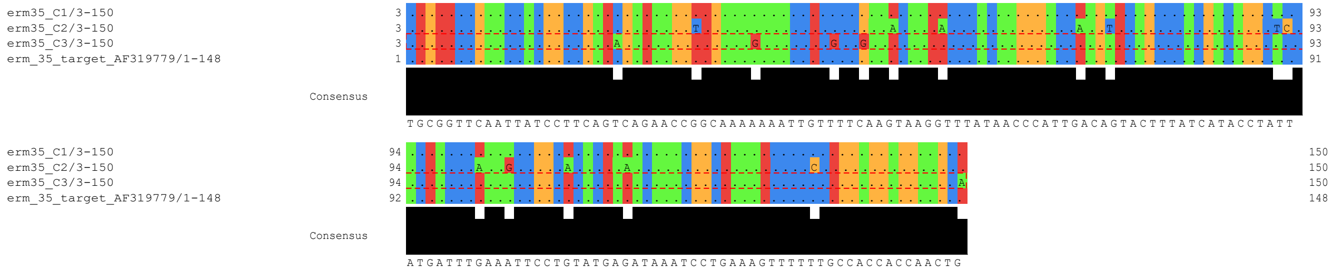

B. *tetM* alignment

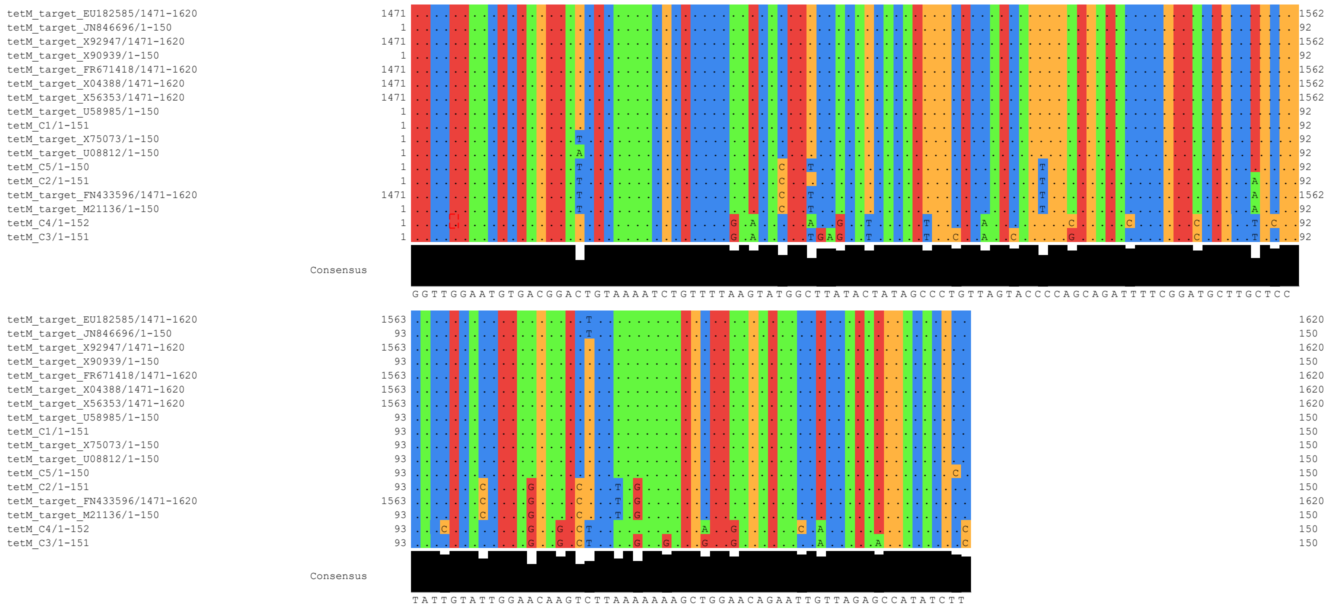

Supp Figure 3. Alignment of gene targets and sequences identified by DARTE-QM gene target for a) *erm35* and b) *tetM* genes. Sequences are representative sequences identified for clusters of reads by 97% sequence identity (see also Supp. Data7). The presence of gene variants are shown in the corresponding three clusters for *erm35* (erm35_C1-C3) and five clusters for *tetM* (tetM_C1-C5). Genes targeted by DARTE-QM are also shown. Start and end basepair coordinates are shown for the alignment of each gene reference.
